# EWS-FLI1-mediated tenascin-C expression promotes tumour progression by targeting MALAT1 through integrin α5β1-mediated YAP activation in Ewing sarcoma

**DOI:** 10.1038/s41416-019-0608-1

**Published:** 2019-10-25

**Authors:** Shaohui He, Quan Huang, Jinbo Hu, Lei Li, Yanbin Xiao, Hongyu Yu, Zhitao Han, Ting Wang, Wang Zhou, Haifeng Wei, Jianru Xiao

**Affiliations:** 1 Spinal Tumor Center, Department of Orthopaedic Oncology, Changzheng Hospital, Second Military Medical University, Shanghai, 200003 P. R. China; 2 Shanghai Key Laboratory of Regulatory Biology, Institute of Biomedical Sciences, School of Life Sciences, East China Normal University, Shanghai, 200241 P. R. China; 3 Department of Orthopaedics, Musculoskeletal Tumor Center of Yunnan Province, the Third Affiliated Hospital of Kunming Medical University, Kunming Medical University, Kunming, 650106 Yunnan P. R. China; 4 Department of Pathology, Changzheng Hospital, Second Military Medical University, Shanghai, 200003 P. R. China; 5 School of Medicine and Life Sciences, Nanjing University of Chinese Medicine, Nanjing, 210023 Jiangsu P. R. China; 6 School of Medicine, Tsinghua University, Beijing, 100084 P. R. China

**Keywords:** Cell biology, Bone cancer

## Abstract

**Background:**

The extracellular matrix has been critically associated with the tumorigenesis and progression of Ewing sarcoma (ES). However, the regulatory and prognostic roles of tenascin-C (TNC) in ES remain unclear.

**Methods:**

TNC expression was examined in specimens by immunohistochemistry, and the association of TNC expression with ES patient survival was also analysed. TNC-knockout cell lines were constructed using CRISPR/Cas9 methods. In vitro experiments and in vivo bioluminescent imaging using BALB/c nude mice were conducted to evaluate the effect of TNC on ES tumour progression. RNA sequencing was performed, and the underlying mechanism of TNC was further explored.

**Results:**

TNC was overexpressed in ES tissue and cell lines, and TNC overexpression was associated with poor survival in ES patients. TNC enhanced cell proliferation, migration and angiogenesis in vitro and promoted ES metastasis in vivo. The oncoprotein EWS-FLI1 profoundly increased TNC expression by directly binding to the TNC promoter region. Metastasis-associated lung adenocarcinoma transcript 1 (MALAT1) upregulation induced by Yes-associated protein (YAP) activation was responsible for TNC-regulated ES tumour progression. Activated integrin α5β1 signalling might be correlated with YAP dephosphorylation and nuclear translocation.

**Conclusions:**

TNC may promote ES tumour progression by targeting MALAT1 through integrin α5β1-mediated YAP activation.

## Background

Ewing sarcoma (ES), the second most common malignant paediatric tumour of bone and soft tissue, shows a high metastatic incidence and can occur at any age but peaks in individuals at 10–20 years old.^1,2^ Despite advanced multidisciplinary treatments for ES, the estimated 2-year survival rate for all patients has remained approximately 52.4%.^3^ Notably, ES is identified by chromosomal translocations that encode members of the EWS-ETS fusion oncoprotein family, of which the EWS-FLI1 oncoprotein is the most common type, accounting for 85% of ES cases.^4,5^ EWS-FLI1 is crucial to establish oncogenic regulation in ES survival, differentiation, and progression.^6,7^

Tenascin-C (TNC) is an extracellular matrix (ECM) glycoprotein that is highly expressed in many kinds of human cancer.^8^ Emerging evidence reveals that TNC promotes tumour pathogenesis and progression through various signalling pathways including integrin signalling,^9–12^ the Hippo signalling pathway,^9,13^ Notch signalling,^14,15^ and the Wnt pathway.^1,14,16^ Moreover, metastasis-associated lung adenocarcinoma transcript 1 (MALAT1), one of the most critical long noncoding RNAs (lncRNAs), is closely implicated in tumour-associated cell behaviours.^17^ MALAT1 overexpression promotes tumour cell proliferation, migration, angiogenesis, and metastasis by a series of mechanisms including chromatin and genomic modification, transcriptional and posttranscriptional regulation, and affecting protein function.^18^

Although increasing evidence demonstrates that TNC and MALAT1 function in tumour behaviours, their connection and underlying mechanisms in regulating ES tumour progression remain to be elucidated. In the current work, we discovered the following: (1) the expression of TNC in ES tumour tissue and cells, (2) the prognostic role of TNC in predicting ES patient survival, (3) the pro-tumorigenic role of TNC in ES cell lines (in vitro) and nude mice (in vivo), (4) the transcriptional role of the EWS-FLI1 oncoprotein in promoting TNC expression, (5) the expression of MALAT1 in ES specimens, and (6) the connection between TNC and MALAT1 and their underlying mechanism in promoting ES tumour progression. To the best of our knowledge, this is the first report that TNC promotes ES tumour progression by upregulating MALAT1 through integrin α5β1-mediated YAP activation. These findings reveal that TNC may serve as a prognostic and therapeutic candidate in ES.

## Methods

### ES specimens and clinical follow-up

Fifty-two ES specimens and thirty-seven adjacent normal tissue specimens were collected after approval by the hospital institutional review boards. This study was carried out in accordance with the Declaration of Helsinki, and written informed consent was obtained from all participants. The general characteristics and outcomes of ES patients are illustrated in Table 1. Metastasis-free survival (MFS) was defined as the period from the first day after operation to the date of metastasis confirmation or October 31^st^, 2018. The overall survival (OS) was defined as the period from the first day after surgery until the date of disease-related death or October 31st, 2018.

**Table 1:** The general information of ES patients

Age (ys)	24.7 ± 13.4 (5–61)
≤10	3 (5.8%)
10–20	20 (38.5%)
>20	29 (55.7%)
Sex	
Male	35 (67.3%)
Female	17 (32.7%)
Tumour location	
Cervical	12 (23.1%)
Thoracic	8 (15.4%)
Lumbar	13 (25.0)
Sacral	11 (21.2%)
Others	8 (15.4%)
Chemotherapy	
Yes	50 (96.2%)
No	2 (3.8%)
Follow up (M)	21.9 ± 13.3 (3.0–68.0)
≤12	11 (21.2%)
12–24	29 (55.7%)
>24	12 (23.1%)
Metastasis	
Yes	38 (73.1%)
No	14 (26.9%)
Dead of disease	
Yes	35 (67.3%)
No	17 (32.7%)

### Immunohistochemistry (IHC) and western blotting

Paraffin-fixed tissue sections were stained via IHC to analyse the expression of TNC and CD99. Incubation with primary antibodies and horseradish peroxidase (HRP)-coupled secondary antibody was performed according to recommendations. The results of IHC were evaluated by experienced pathologists in a blinded manner.

Cells were lysed in NP40 cell lysis buffer (Invitrogen) containing a 1:1000 proteinase inhibitor cocktail (Biotool) according to the manufacturer’s recommendations. After standard quantification, separation, and membrane transfer, the immunoperoxidase method was utilised with SuperSignal^TM^ West Pico PLUS Chemiluminescent substrate (Thermo Scientific) to detect proteins. All the primary and secondary antibodies used are listed in Supplementary Table 1.

### Cell culture

ES cell lines (A673 [CVCL_0080] and SKNMC [CVCL_0530]) and human bone marrow stromal cells (BMSCs, isolated from normal donors as previously described^19^) were cultured in RPMI 1640 medium (Gibco) containing 10% foetal bovine serum (FBS, Gibco) and 100 U/ml penicillin/streptomycin (P/S, Gibco). Human umbilical vein endothelial cells (HUVECs [CVCL_2959]) and HEK293 cells (CVCL_0045) were grown in EGM-2 medium (Lonza) and DMEM (HyClone) with 10% FBS, respectively. Cultures were maintained in a 37 °C incubator in 5% CO_2_. The purity and authenticity of the cell lines were assessed via short tandem repeat profiling. Cells were tested regularly to ensure they were free of mycoplasma infections. Cells were cultured to a proper density and sub-cultured every 3–4 days according to the methods of a previous study.^7^ Exogenous recombinant TNC was purchased from Abcam. Verteporfin and saracatinib were purchased from Selleckchem.

### CRISPR/Cas9-mediated TNC knockout (ko)

For TNC-ko experiments, a CRISPR/Cas9-sgRNA genome-wide library (CRISPR-Pool™ KOUT, GeneChem) was used according to previous studies.^20,21^ The targeted sequence was GTTGCCCCGACCGCTACAGA. Generally, the viral vector was co-transfected with pVSVg and psPAX2 into HEK 293T cells with Lipofectamine 2000 (Invitrogen). After 48–72 h of co-transfection in DMEM, the viral supernatant was collected, filtered through a 0.45 µm filter, and then ultra-centrifuged at 25,000 rpm for 2 h at 4 °C. Quality tests were performed to confirm the viral titre without contamination. Then, viral pellets were resuspended in RPMI 1640 medium and added to A673 and SKNMC cell cultures for 24 h, followed by puromycin (Gibco) selection for seven days. Stable cell lines were verified by Sanger sequencing and western blotting analysis at the gene and protein levels, respectively.

### qRT-PCR

Total RNA was extracted using TRIzol (Invitrogen) and reverse transcribed into cDNA with PrimeScript™ RT Master Mix (TaKaRa). Different gene transcripts were quantified on a 7900HT Fast Real-Time PCR System (Life Technologies) with SYBR Premix Ex Taq™ II (TaKaRa). The primers used for qRT-PCR in this study are listed in Supplementary Table 2.

### Cell viability and migration assays

Cells were seeded in 96-well plates (5 × 10^3^/well) with or without exogenous TNC and cultured for 48 h. Cell Counting Kit-8 reagent (Dojindo Laboratories) (10 µl/well) was added and incubated for 1.5 h at 37 °C. The absorbance at 450 nm was measured using an ELx800 microplate reader (BioTek Instruments, Inc.). The average absorbance of each sample was calculated from five duplicates.

For the cell migration assay, cells (1 × 10^5^/well) were added to the upper chamber of a Transwell migration chamber, with 0.1% FBS medium and 10% FBS medium loaded into the lower chamber (8 μm pore size Transwell migration chamber, Corning). After 24 h of incubation, cells were washed and fixed with 4% paraformaldehyde for 20 min. Non-invasive cells were removed from the upper compartment with a cotton swab. After crystal violet staining for 15 min, cells that had migrated through the membrane from four random fields in each membrane were counted with Image-Pro Plus 6.0 software.

### Tube formation assay

Matrigel (BD Bioscience) had previously been thawed at 4 °C overnight. Then, 50 µl of chilled Matrigel was seeded in a 96-well plate and incubated at 37 °C for 45 min. HUVECs (1 × 10^4^/well, 70–80% confluence) suspended in 100 µl of EGM-2 medium and conditioned medium from TNC-wild-type (wt) or TNC-ko ES cells were added to the solidified Matrigel. The rescue capacity was simultaneously evaluated in the presence of exogenous TNC. After 18 h of incubation at 37°C, capillary-like structures from five microscopic fields were photographed to evaluate angiogenesis capability.

### Animal experiments and bioluminescence imaging assessment

All animal experiments were approved by the Animal Experimental Committees of the Second Military Medical University and performed in accordance with the Animals (Scientific Procedures) Act 1986^22^ and the updated guidelines. All animal studies were reported according to the ARRIVE guidelines for reporting experiments involving animals. Four- to six-week-old BALB/c male nude mice with similar weights (16 ± 2 g) were purchased from the SLAC Laboratory (Shanghai, China) and housed in the animal room of the SPF laboratory under standard conditions. The mice were randomly divided into two groups (eight mice per group, four mice per cage). A total of 1 × 10^6^ luciferase-labelled A673 cells (TNC-wt & TNC-ko) were digested, resuspended in 100 μl of PBS, and then injected into mice via the tail vein to study the in vivo effect of TNC on metastasis. In vivo metastatic colonisation was measured every three to five days from day 0 to day 28 (the mice were sacrificed with carbon dioxide on day 28.) with the IVIS Spectrum in vivo imaging system (PerkinElmer, Inc.).^14,23^ Briefly, the mice were anaesthetised (using isoflurane inhalation) and intraperitoneally injected with 150 mg/kg D-luciferin potassium salt (AOK Chem). After 10 min, the mice were imaged and assessed using Living Image 3.2 software (Caliper). The animal study was performed and assessed by two different investigators to minimise subjective bias.

### Luciferase reporter assay

Recombinant plasmid was established from the synthesised promoter region of TNC (nt −2000~0) (TsingKe) and the linearised pGL3-basic plasmid using a Quick-Fusion Cloning Kit (Biotool). The EWS-FLI1 and FLI1 overexpression plasmids were also constructed using the kit above. Dual-luciferase assays were conducted in a 24-well plate. The recombinant plasmid and phRL-TK100 Renilla reporter plasmid, together with the EWS-FLI1 or FLI1 overexpression plasmids, were transfected into 60–70% confluent HEK293 cells. After 48 h of co-transfection, a dual-luciferase assay kit (Promega) was used to quantify firefly and Renilla luciferase activities based on the manufacturer’s instructions.

### Chromatin immunoprecipitation (ChIP) assay

ChIP assays were conducted with a ChIP assay kit (Millipore) according to the manufacturer’s instructions. Formaldehyde (1%) was used to crosslink the chromatin in A673 cells (10 min) at room temperature, and glycine was generated to terminate the crosslinking. The lysis buffer (with SDS added) was then sonicated to generate 200 to 500 bp DNA fragments. Anti-FLI1 antibody was incubated with the DNA fragments, which were subjected to ChIP assays. Normal IgG was used as a nonspecific control. Then, the eluted DNA was enriched and investigated by PCR. The primers used in the ChIP assay are listed in Supplementary Table 3.

### RNA extraction and differential gene expression profiling

Total RNA was extracted from A673 cells (TNC-wt & TNC-ko) using TRIzol (Invitrogen). The purity of the RNA was determined using a kaiaoK5500^®^ spectrophotometer (Beijing, China). The RNA integrity and concentration were assessed via a Bioanalyzer 2100 system with an RNA Nano 6000 Assay Kit (Agilent Technologies). Sequencing libraries were established using the NEBNext® Ultra™ Directional RNA Library Prep Kit for Illumina® (NEB, Inc.). After regular examination, libraries were sequenced on an Illumina HiSeq X Ten platform with 150 bp paired-end reads. Differential gene expression was analysed by DEGseq v.1.18.0. Statistical comparisons were performed by analysis of variance.

### Fluorescence in situ hybridisation (FISH) and Immunocytochemistry (ICC)

The relevant antibodies are listed in Supplementary Table 2. Expression of the lncRNA MALAT1 was detected in 5 μm unstained ES tissue sections via FISH^24^ with human MALAT1 with CAL Fluor® Red 610 dye (Stellaris® FISH Probes, LGC Biosearch Technologies). ICC was performed to detect YAP expression in ES cells using immunofluorescence (IF) staining.^9^ The FISH and IF signals were monitored by two independent investigators with a BX51 fluorescence microscope (Olympus).

### Statistical analysis

SPSS v.19.0 software and GraphPad Prism v.6.01 software were used for statistical analysis. Quantitative data are described as the mean/median (standard deviation) and were compared by Student’s t-test and ANOVA with Tukey’s multiple comparisons test when appropriate. Qualitative data are reported as counts/percentages and were compared with the chi-square test or Fisher’s exact test when appropriate. Correlation analysis was performed by using Spearman rank correlation analysis. Survival probability was estimated by the Kaplan–Meier method, and log-rank tests were used to compare metastasis-free survival (MFS) and overall survival (OS) between groups. All in vitro experiments were repeated three times independently with at least three replicates per experiment, and representative experiments are shown. Differences with *p* values < 0.05 were considered statistically significant (*p*-values: *p < 0.05; **p < 0.01; ***p < 0.001).

## Results

### TNC overexpression in specimens is associated with ES patient survival

**Fig. 1:**
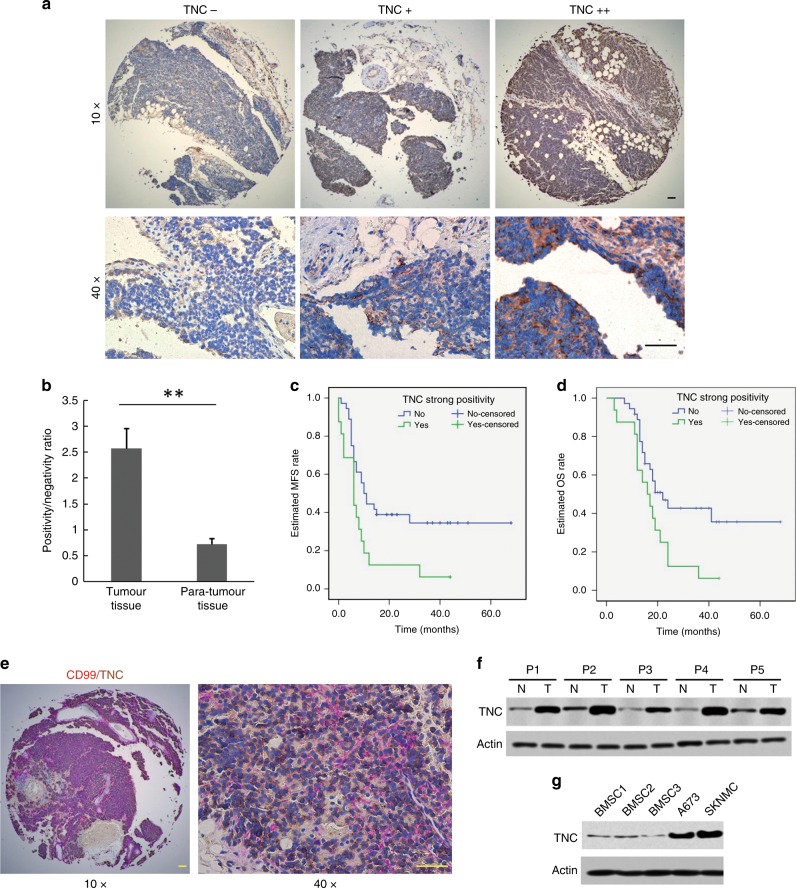
TNC expression in ES tissues and cell lines. **a** Representative TNC expression in ES samples measured by IHC (×10 and ×40). **b** Relative TNC expression (positive/negative ratio) in tumour tissues (*n* = 52) and para-tumour normal tissues (*n* = 37) (*p* < 0.01), with the results evaluated by two independent investigators. **c**, **d** Kaplan–Meier curves based on TNC expression showing MFS (*p* = 0.012) and OS (*p* = 0.02) (No/Yes indicates whether the IHC result was not or was, respectively, strongly TNC-positive). **e** Double-labelling IHC indicating the co-expression of TNC (brown) and CD99 (purple) in ES tissue (10× and 40×). **f** Confirmation of TNC overexpression in tumour tissues by western blotting. **g** Western blotting confirmed TNC overexpression in A673 and SKNMC cells (scale bars, 150 μm)

TNC overexpression was observed in clinical ES tissues (×10 and ×40) and compare to that in adjacent normal tissue via IHC staining (Fig. 1a). The IHC results were assessed by two investigators independently, and thirty-seven (71.2%) specimens tested positive for TNC compared to sixteen (43.2%) para-tumour tissue specimens that tested positive for TNC. The difference in TNC expression (positive/negative ratio) between tumour and adjacent normal tissue was statistically significant (*p* < 0.01) (Fig. 1b). As shown in Supplementary Table 1, the mean follow-up time of the enrolled patients was 21.9 ± 13.3 months (range 3.0–68.0 months). Thirty-eight (73.1%) patients developed metastasis, and 35 (67.3%) patients died because of the disease. The estimated MFS and OS rates were 25.2 and 24.6%, respectively, according to the Kaplan–Meier method. The mean MFS and OS were 23.3 ± 3.8 and 30.0 ± 3.5 months, respectively. IHC revealed that the percentage of specimens strongly positive for TNC was 30.8% (*n* = 16). When analysing the follow-up data, the log-rank test was further used to evaluate the impact of TNC positivity on patient disease progression and overall survival. The Kaplan–Meier curve showed that strong positivity for TNC was correlated with a worse MFS (*p* = 0.012) (Fig. 1c), and ES patients with strong positivity for TNC also had a worse OS (*p* = 0.02) than those who were not strongly positive for TNC (Fig. 1d).

Moreover, since CD99 is a specific marker for ES found in the cytoplasmic membrane of 100% ES tumour cells,^25,26^ the IHC double-staining method was applied to detect the co-expression of TNC and CD99 in ES tissue. TNC (brown) and CD99 (purple) were co-expressed at high levels in ES tissue under ×10 and ×40 magnification (Fig. 1e). TNC overexpression in ES tissue and cells was then confirmed using western blotting (Fig. 1f, g).

### TNC depletion inhibits ES cell proliferation, migration and angiogenesis

**Fig. 2:**
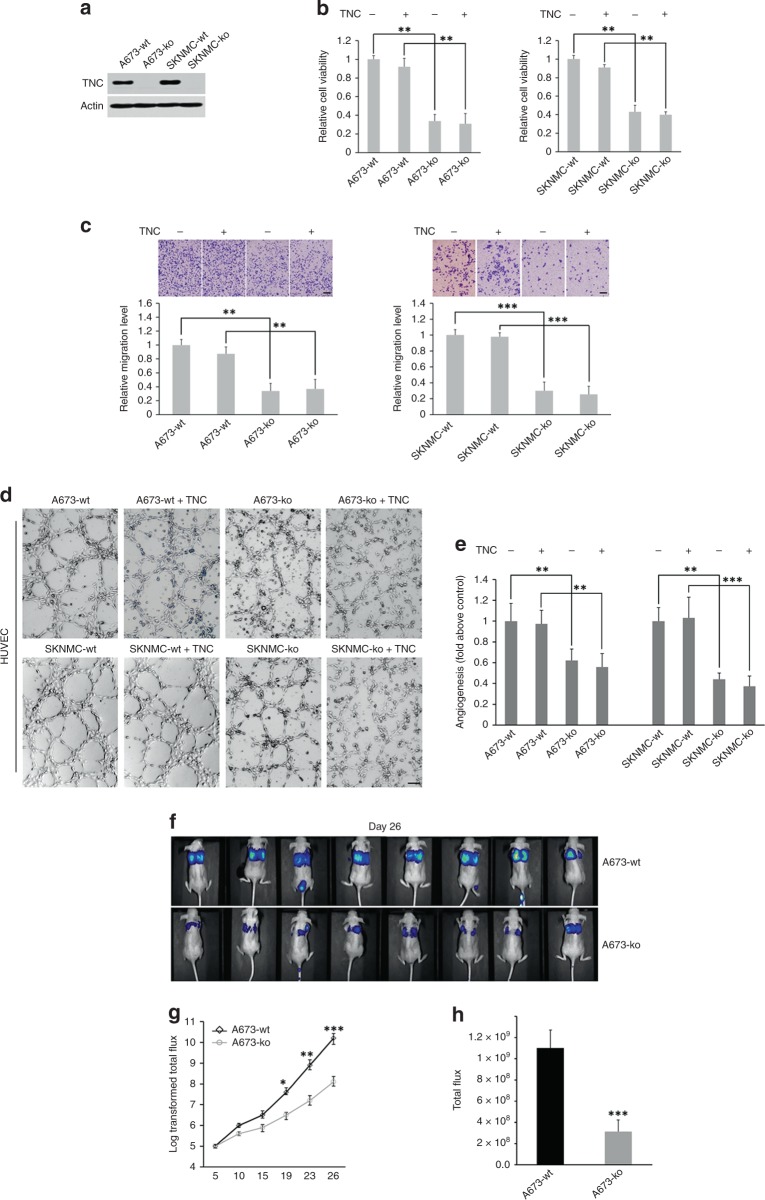
TNC-induced enhanced ES cell proliferation, migration and angiogenesis were confirmed in vitro, and TNC-induced ES cell metastasis was confirmed in vivo. **a** TNC stable knockout A673 and SKNMC cell lines were established and confirmed by western blotting. **b**, **c** TNC-induced enhancement of ES cell (A673 and SKNMC cells) viability and migration, respectively (*n* = 4 wells per group). **d**, **e** Representative images and statistical analysis of TNC-induced promotion of angiogenesis (A673 and SKNMC cells, *n* = 3 wells per group). **f** Representative bioluminescence images at day 26. **g** In vivo bioluminescence images showing statistically significant differences 19 days after inoculation, with this difference peaking on day 26 (*n* = 8 mice per group). All in vitro experiments were repeated three times independently with at least three replicates per experiment (scale bars, 100 μm)

TNC-ko cell lines were initially established using the CRISPR/Cas9 genome editing technique as described above.^20,21^ After puromycin selection for seven days, clones were generated by multiple proportion dilution and cultured in 96-well plates. DNA was extracted when cells in 24-well plates were 90–100% confluent and sequenced using the Sanger method. A frameshift mutation around “AGG” in both A673 and SKNMC cells was confirmed (Supplementary Fig. 1), and the knockdown of TNC expression was further validated via western blotting (Fig. 2a).

A CCK8 assay to assess A673 and SKNMC cell viability showed that the proliferation rates of the TNC-ko groups were significantly lower than those of the TNC-wt groups (*p* < 0.01) and that the addition of exogenous TNC failed to rescue tumour cell growth in both the TNC-wt and TNC-ko groups (Fig. 2b). As shown by migration experiments, the knockout of TNC inhibited ES migration in both A673 (*p* < 0.01) and SKNMC cells (*p* < 0.001) (Fig. 2c). However, the addition of exogenous TNC had no significant impact on the migration capacity of A673 and SKNMC cells. This is probably due to the use of recombinant TNC (amino acids 181–290) instead of full-length purified TNC.^10^

Due to the pivotal role of the blood supply in tumorigenesis, we further performed tube formation experiments to evaluate the role of TNC in ES-induced angiogenesis. The knockout of TNC impaired angiogenesis compared with the TNC-ko groups, but the difference in capillary-like structures after the addition of exogenous TNC was no different between the TNC-ko and TNC-wt groups (in both A673 and SKNMC cells). Representative images and relative statistics are illustrated in Fig. 2d, e.

### Metastasis enhancement is driven by TNC

The metastatic colonisation abilities of the TNC-wt and TNC-ko groups (A673) determined by bioluminescence imaging were compared in in vivo experiments. The mice in our study had similar weights before experiments, and no mouse died due to severe adverse effects during the experiments. The mean total flux of the mice (*n* = 8 per group) injected with TNC-wt cells was higher than that of mice injected with TNC-ko cells 19 days after injection (*p* < 0.05), and this difference was highest at day 26 after inoculation (*p* < 0.001). Representative bioluminescence images at day 26 and growth curves are shown in Fig. 2f–h.

### EWS-FLI1 significantly activates TNC expression in ES cells

**Fig. 3:**
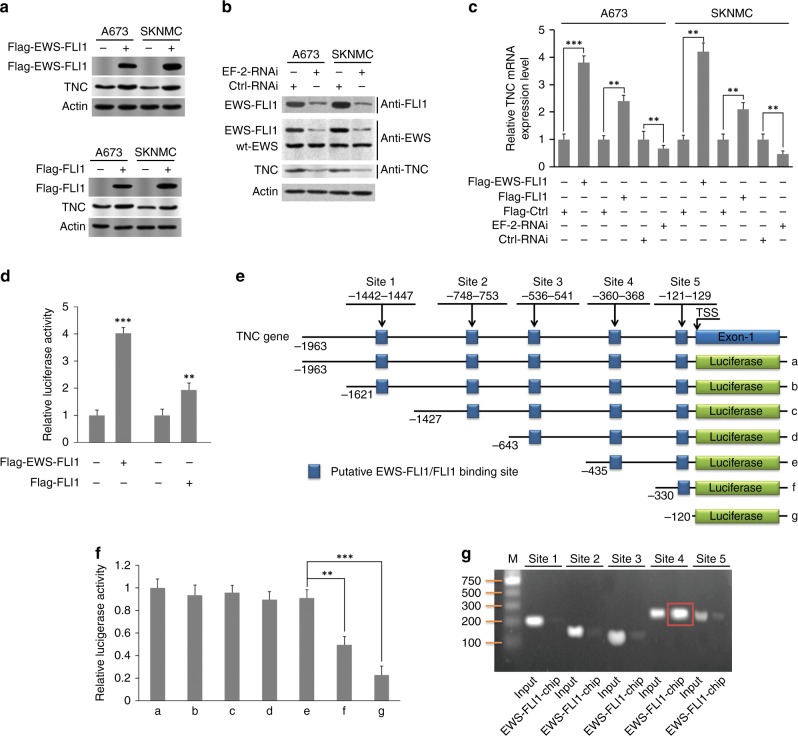
Promotion of TNC expression driven by EWS-FLI1. **a** TNC overexpression was induced by EWS-FLI1 and FLI1 in A673 and SKNMC cells, respectively. **b** EWS-FLI1 expression was knocked down by transfection with siRNA that encoded EF-2-RNAi sequences (as detected by both anti-FLI1 and anti-EWSR/EWS antibodies), and TNC deficiency was detected EWS-FLI1 silencing. **c** Statistically significant differences in TNC mRNA expression were found between the EWS-FLI1-overexpressed vs. ctrl groups (*p* < 0.001 for A673 cells, *p* < 0.001 for SKNMC cells), FLI1-overexpressed vs. ctrl groups (*p* < 0.01), and EWS-FLI1-deficient vs. ctrl groups (*p* < 0.01). **d** Luciferase activity was measured between the groups above (*n* = 3 replicates per group). **e**, **f** Putative EWS-FLI1/FLI1-binding sites in the TNC promoter region (*n* = 3 replicates per group). **f** ChIP assay indicated that site 4 (−360~−368) is the probable binding site for the interaction of EWS-FLI1 with TNC. Representative data from one of three independent experiments are shown

Flag-EWS-FLI1 and Flag-FLI1 plasmids were transfected into ES cells, and the overexpression of both EWS-FLI1 and FLI1 was found to promote TNC expression according to immunoblotting analysis (A673 and SKNMC cells, Fig. 3a). EWS-FLI1 expression was knocked down by transfecting cells with siRNA encoding EF-2RNAi and Ctrl-RNAi sequences (referring to a previous study^27^). We then used anti-FLI1 and anti-EWS antibodies to detect EWS-FLI1 expression and found that anti-FLI1 could detect the EWS-FLI1 fusion oncoprotein without FLI1, while anti-EWS could detect both the EWS-FLI1 and EWS proteins. Consistent TNC downregulation was confirmed in both A673 and SKNMC cells (Fig. 3b). qRT-PCR was then performed to detect TNC mRNA expression, and the differences in TNC mRNA expression between the EWS-FLI1-overexpressed vs. ctrl groups (*p* < 0.001 in A673 cells, *p* < 0.001 in SKNMC cells), FLI1-overexpressed vs. ctrl groups (*p* < 0.01), and EWS-FLI1-deficient vs. ctrl groups (*p* < 0.01) were statistically significant (Fig. 3c). Meanwhile, a dual-luciferase reporter assay revealed higher relative luciferase activities in the presence of EWS-FLI1 (*p* < 0.001) or FLI1 (*p* < 0.01) compared with those in the control groups (A673 and SKNMC cells). Moreover, EWS-FLI1 seemed to have a higher affinity for the TNC promoter than FLI1 did (Fig. 3d). To determine whether TNC is transactivated by EWS-FLI/FLI, we searched for the EWS-FLI/FLI consensus sequence in the promoter region of the TNC gene from 2 kb upstream to exon 1. We identified five putative binding sites and examined the relative luciferase activities at different sites with a series of luciferase reporter plasmids (Fig. 3e). We built the different recombinant plasmids by inserting the synthesised promoter region into the pGL3-basic plasmid, and TK100 plasmids were transfected together as an internal standard for transfection efficiency normalisation. TNC promoter activity was greatly enhanced by the region encompassing nt −360~−368 (site 4) (*p* < 0.01) and nt −121~−129 (site 5) (*p* < 0.001) (Fig. 3f), which indicated that EWS-FLI1/FLI1 might promote TNC expression via binding to sites 4 and 5 of the TNC promoter. A ChIP assay was also performed to further confirm the interaction between EWS-FLI1 and TNC, and the signal for site 4 was higher than that for site 5; thus, site 4 might be the EWS-FLI1-binding site in the TNC promoter (Fig. 3g).

### MALAT1 is overexpressed in ES and associated with TNC

**Fig. 4:**
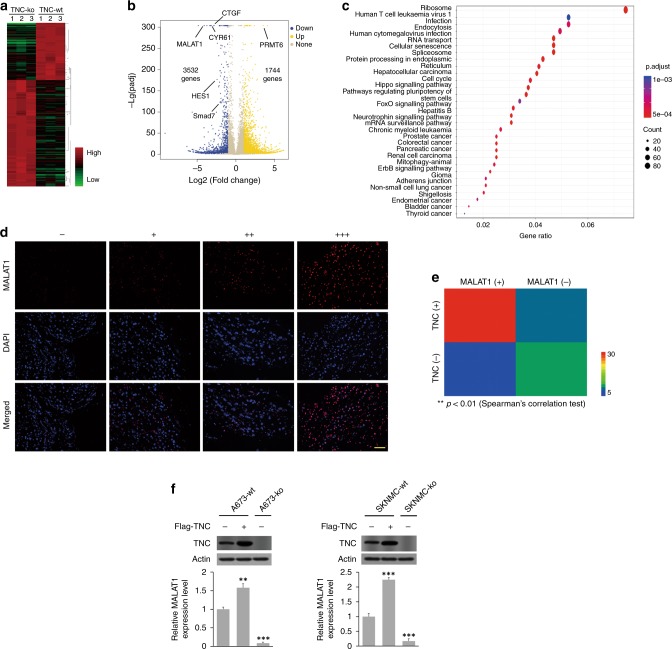
MALAT1 upregulation induced by TNC. **a**, **b** Differential gene expression is delineated in a heat map (normalised counts >10, ∣log_2_ fold change ∣> 1.5) and volcano plot (*n* = 3 replicates per group, A673 cells). **c** Enriched KEGG pathways were determined by using clusterProfiler R package version 3.4.1. **d**, **e**,Representative images and statistical analysis of MALAT1 expression in ES clinical specimens (scale bars, 100 μm). **f** Relative MALAT1 relative mRNA expression compared with the wild-type group was upregulated by transfection with Flag-TNC plasmid (*p* < 0.01 in A673 cells, *p* < 0.001 in SKNMC cells), and downregulated dramatically by TNC depletion (*p* < 0.001) (normalised to GAPDH expression)

RNA-seq was performed in TNC-wt and TNC-ko A673 cells, and the results were compared to explore the possible downstream genes regulated to TNC. Differential gene expression was delineated with a heat map (Fig. 4a). According to the volcano plot, 3532 genes were upregulated, and 1744 genes were downregulated. Among the differentially expressed genes, MALAT1 expression differed by >16-fold between groups and was one of the most statistically significant differentially expressed genes. KEGG pathway enrichment analysis revealed that the mechanism by which TNC regulates ES tumour progression might be correlated with Hippo signalling pathway (Fig. 4c). We detected and compared MALAT1 expression in ES tissue and adjacent normal tissue by using a fluorescent-labelled oligonucleotide probe. The results showed prevalent MALAT1 overexpression in ES tissue with respect to normal tissue (Fig. 4d). By combining the TNC expression results, we found that MALAT1 was positively expressed in thirty-three specimens, of which twenty-eight were TNC-positive (including sixteen specimens with strong positivity and twelve specimens with moderate positivity). Ten of the remaining nineteen specimens were negative for both MALAT1 and TNC expression. The Spearman chi-square test was further used to analyse the correlation between MALAT1 and TNC expression, and the heat map revealed the robust correlation between MALAT1 and TNC expression in ES specimens (*p* < 0.01) (Fig. 4e). Further experiments implied that the overexpression of TNC can promote MALAT1 expression in A673 (*p* < 0.01) and SKNMC (*p* < 0.001) cells, and the difference in MALAT1 expression between the TNC-wt and TNC-ko cell lines was statistically significant (*p* < 0.001). These results indicated that MALAT1 is associated with TNC-regulated ES tumour progression.

### TNC may regulate MALAT1 through integrin α5β1-mediated YAP activation

**Fig. 5:**
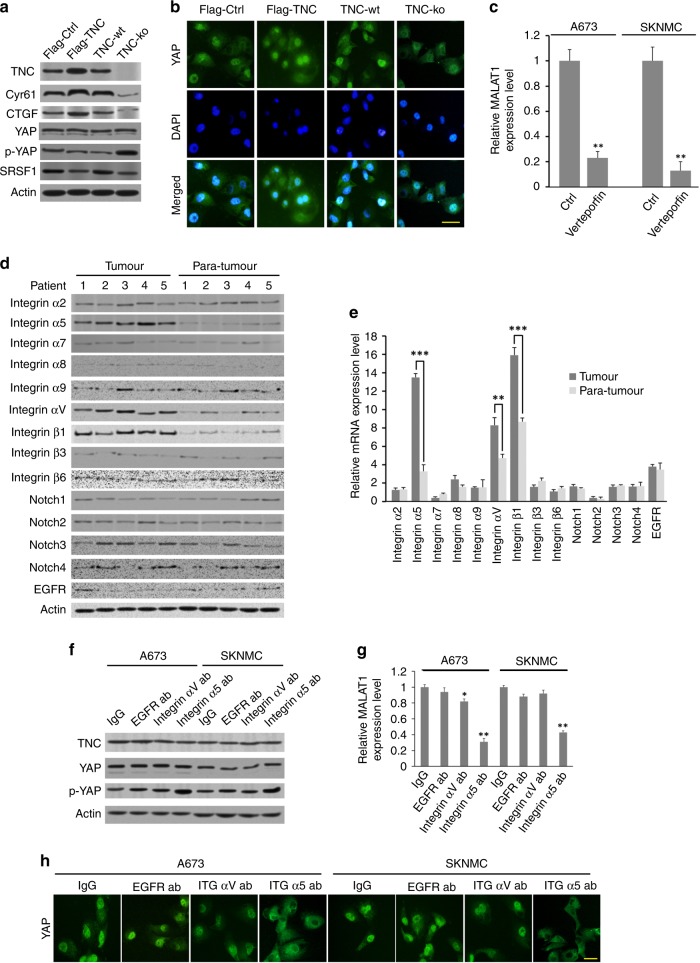
The mechanism by which TNC regulates MALAT1 overexpression. **a** Effect of TNC on key factors and expression of downstream genes in the Hippo signalling pathway in A673 cells. **b** IF staining showing YAP subcellular localisation in TNC-overexpressing, TNC-wt, and TNC-ko cells (A673 cells). **c** Effect of the YAP inhibitor verteporfin on MALAT1 expression in A673 and SKNMC cells (*n* = 3 replicates per group). **d**, **e** The protein and mRNA expression of TNC-associated receptors in tumour and para-tumour specimens. **f**, **g** Effect of TNC-associated receptor blockade on YAP and MALAT1 expression in both A673 and SKNMC cells (*n* = 3 replicates per group in **g**). **h** Representative IF staining showing YAP subcellular localisation following blockade of TNC-associated receptors in both A673 and SKNMC cells (scale bars, 20 μm)

Further analysis showed that the expression of key downstream target genes of the Hippo signalling pathway (CTGF and CYR61) was downregulated in TNC-ko cells compared to TNC-wt cells (Fig. 4b). Since YAP, a key transcriptional cofactor in the Hippo signalling pathway, has been reported to regulate MALAT1 expression by binding to the MALAT1 promoter,^17,28^ we investigated whether TNC upregulates MALAT1 through YAP. Although the total YAP expression levels were unchanged by TNC expression, phosphorylated YAP levels in A673 cells were decreased, and CTGF and CYR61 expression levels were positively correlated with the TNC expression level. In addition, the YAP inhibitor serine/arginine-rich splicing factor 1 (SRSF1) was downregulated regardless of TNC overexpression or depletion (Fig. 5a). Furthermore, IF staining revealed that YAP nuclear localisation was decreased in TNC-ko A673 cells compared to TNC-wt A673 cells, while TNC-overexpressing cells exhibited enhanced YAP nuclear translocation compared to TNC-wt cells (Fig. 5b). Meanwhile, MALAT1 expression in A673 cells was decreased significantly after 48 h of pharmacological administration of verteporfin, a potent YAP inhibitor (*p* < 0.01) (Fig. 5c).

Subsequently, western blotting for TNC-associated receptors showed the increased expression of integrins α5, αV, and β1 with respect to normal tissue (Fig. 5d), but no obvious differences in integrins α2, α7, α8, α9, β3, and β6; Notch 1, 2, 3, and 4; or EGFR expression were found. The qRT-PCR results also indicated a high level of integrin α5 and integrin β1 mRNA expression (*p* < 0.001), followed by integrin αV mRNA expression (*p* < 0.01) (Fig. 5e). Further experiments revealed that the blockade of integrin α5 using monoclonal antibody increased YAP phosphorylation in both A673 and SKNMC cells, while the inhibition of integrin αV or EGFR did not influence the phosphorylated status of YAP compared with the control group (Fig. 5f). Then, qRT-PCR showed that MALAT1 expression was downregulated significantly when integrin α5 was blocked in both ES cell lines (*p* < 0.01), and antibody against integrin αV caused a decrease in MALAT1 expression in only A673 cells (*p* < 0.05) (Fig. 5g). Moreover, unlike the IgG isotope control, marked YAP cytoplasmic retention was observed after the blockade of integrin α5 but not integrin αV or EGFR (Fig. 5h).

**Fig. 6:**
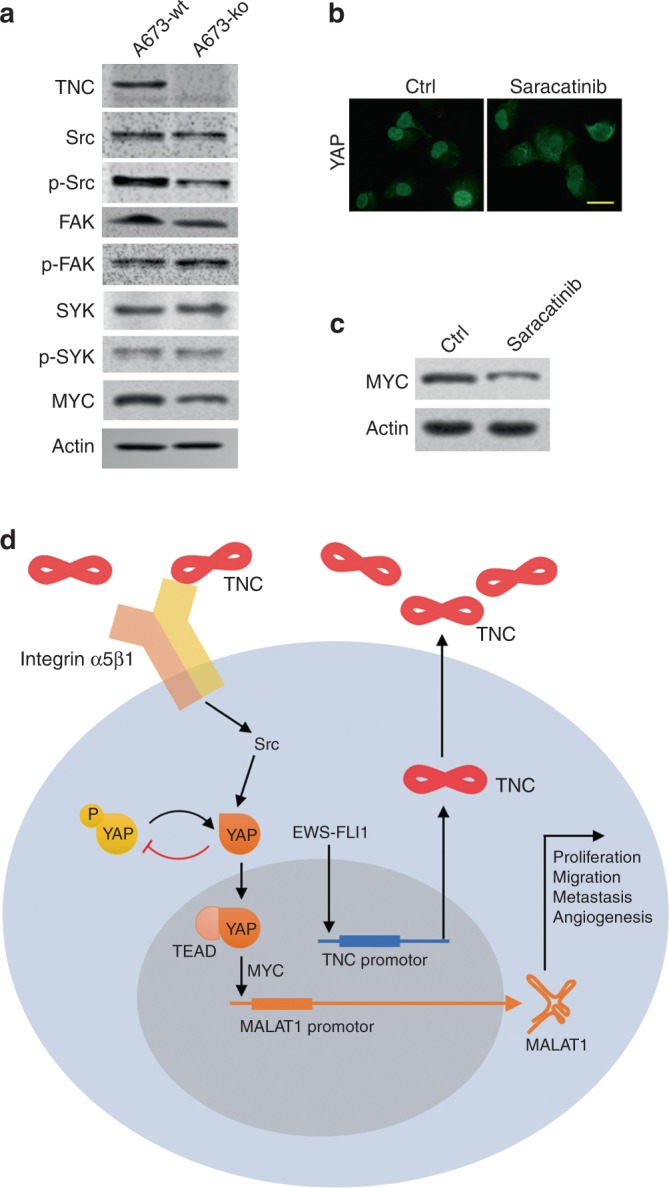
Study on the mechanism by which TNC regulates ES and the prognostic value of TNC in ES. **a** Expression levels of total and phosphorylated kinases and MYC in TNC-wt and TNC-ko A673 cells. **b** Effect of an Src inhibitor (saracatinib) on YAP subcellular localisation in A673 cells determined via ICC (scale bars, 20 μm). **c** Effect of an Src inhibitor (saracatinib) on MYC expression. **d** Schematic diagram showing the role of TNC in regulating ES tumour progression. Representative data from one of three independent experiments are shown

We further detected the expression of kinases downstream of integrin by western blotting. The levels of phosphorylated Src and v-myc myelocytomatosis viral oncogene homologue (MYC) were significantly decreased in TNC-ko A673 cells compared to those in TNC-wt A673 cells, while the level of phosphorylated focal adhesion kinase (FAK) and spleen tyrosine kinase (SYK) seemed not to have changed (Fig. 6a). Meanwhile, pharmaceutical administration of a potent Src inhibitor (saracatinib) resulted in the prominent inhibition of YAP nuclear translocation, as shown by IF (Fig. 6b), and decreased MYC expression, as shown by immunoblotting (Fig. 6c), in contrast with those control A673 cells. These results implied that Src activation is partly responsible for integrin α5β1-mediated YAP activation and that MYC is partly responsible for YAP-mediated MALAT1 upregulation.

## Discussion

ECM proteins are pivotal in regulating tumorigenesis and progression in most tumour types through matrix-cell interactions.^15^ TNC is one of the most prominent ECM proteins, and the overexpression of TNC is associated with many aspects of tumour cell behaviours in various tumours.^8^ However, the regulatory role of TNC and the underlying mechanism of this role in ES remain obscure. In the current study, we demonstrate that the EWS-FLI1 oncoprotein, which is characteristic of ES, enhances TNC expression by directly binding to its promoter region and that integrin α5β1-mediated YAP activation may be responsible for MALAT1 upregulation in TNC-induced ES tumour progression (Fig. 6d).

TNC is overexpressed in ES tissue and cell lines compared to its expression in normal tissue and cell lines. In vivo experiments revealed that TNC may contribute to ES metastasis, which is consistent with the results of a previous study.^1^ Indeed, autocrine TNC expression enables tumour cells to survive as a metastatic niche component at the early stages of colonisation.^14^ Meanwhile, in a clinical study, the presence of metastasis was determined to be the strongest predictor of prognosis, with the overall survival rate decreased from 65% to less than 30% with the presence of metastasis.^5^ Our survival analysis found a robust correlation between TNC expression and ES patient survival, and this correlation was also previously reported in breast cancer and osteosarcoma.^9,14^ These findings imply that TNC expression predicts ES survival and can serve as a potential prognostic marker for ES in clinical practice.

Furthermore, the knockout of TNC inhibited ES cell proliferation, migration, and angiogenesis in vitro, which indicates that TNC promotes ES tumour progression in an autocrine manner. However, the addition of exogenous TNC failed to antagonise this repression in our study. The use of recombinant TNC (amino acids 181–290) but not full-length purified TNC might account for the failure of TNC to rescue these effects.^10^ Notably, we first reported the role of TNC in facilitating ES angiogenesis, which may be related to its regulation of vascular endothelial growth factor (VEGF) expression.^29^ Nonetheless, the dual angio-modulatory role of TNC in glioblastoma was previously reported by Rupp and colleagues because TNC regulates both pro- and anti-angiogenic signalling.^13^ In addition, in vivo imaging revealed that TNC overexpression promoted distant metastases in immunodeficient mouse models, which indicated that ES patients with TNC overexpression may be more likely to develop metastases.

Due to the prevalence of the EWS-FLI1 oncoprotein in ES and its crucial role as an ES biomarker,^5,30^ we first detected the putative role of EWS-FLI1 in promoting TNC transcription through bioinformatics analysis and further explored this role by the dual-luciferase reporter and ChIP assays. EWS-FLI1 profoundly promoted TNC expression by putatively binding to site 4 (−360~−368) of the TNC promoter, and Watanabe G and colleagues^31^ reported that EWS-ETSs upregulated the TNC gene through four ETS-binding sites between nt −30~−130 in the TNC promoter. This binding indicates that TNC may be closely involved in ES tumorigenesis and at least partly accounts for the EWS-FLI1-mediated oncogenic effect. This finding also provides a possible explanation for TNC overexpression observed in most of the clinical ES tumour tissues. Indeed, TNC overexpression driven by EWS-FLI1 in the tumour-associated microenvironment may play an enhanced role in persistently promoting ES to metastasise to distant organs.^14^

RNA sequencing and FISH revealed MALAT1 overexpression in ES cells and tumour tissues, which implies that MALAT1 is associated with TNC regulation in ES. Although MALAT1 has been proven to be of the most significant lncRNAs that regulates various tumour types,^17,18,32–34^ little information regarding the relationship between TNC and MALAT1 is available. We confirmed the strong correlation between MALAT1 overexpression and TNC in ES tumour tissue. In addition, overexpression of TNC dramatically increased MALAT1 expression, while TNC depletion decreased MALAT1 expression (Fig. 4f). These results collectively indicate that TNC may regulate ES tumour progression by targeting MALAT1.

Moreover, enrichment of the Hippo signalling pathway and the downregulation of its downstream targeted genes (CTGF, CYR61) implies that Hippo signalling is also involved in TNC-mediated regulation of ES. We thus explored the potential role of YAP, one of the key transcriptional coactivators of the Hippo signalling pathway, in regulating ES pathogenesis. The overexpression of TNC markedly decreased YAP phosphorylation and enhanced YAP nuclear localisation, as confirmed by ICC. Dephosphorylated YAP further activates itself and translocates to the nucleus by negatively regulating Hippo signalling.^35^ Activated YAP also transcriptionally activates MALAT1 by binding to a region of the MALAT1 promoter,^17,28^ and this enhanced MALAT1 expression may trigger the expression of downstream genes to positively regulate ES tumour progression.^36^ Consistently, in our study, MALAT1 expression was significantly decreased by the pharmacological inhibition of YAP with verteporfin. Altogether, these results suggest that TNC-induced YAP dephosphorylation and nuclear translocation mediate MALAT1 overexpression and negatively regulate the canonical Hippo signalling pathway.

Further experiments revealed that TNC might regulate YAP-mediated MALAT1 expression by activating integrin α5β1. Integrin family members, including integrins α9β1, αvβ1, α5β1, and αvβ3,^8^ are crucial receptors of TNC that regulate matrix-cell interactions in the cancer microenvironment. Among these integrins, integrin α5β1 is one of the most significant members of the integrin family that influences tumour survival, proliferation, migration, and chemo-resistance.^37–39^ TNC was reported to regulate various cell behaviours by interacting with integrin α5β1 in both a syndecan-4-dependent and syndecan-4-independent manner. For instance, the TNC-integrin α5β1 interaction was shown to block cell adhesion, stimulate tumour cell proliferation,^40^ regulate the chemotaxis of human monocytes and polymorphonuclear leucocytes,^41^ inhibit fibroblast cell cycle progression,^42^ protects cancer stem-like cells from immune surveillance,^43^ and enhance cell survival and proliferation in a platelet-derived growth factor-dependent way.^44^ We further discovered decreased phosphorylation of Src (but not FAK) induced by knockout of TNC and that YAP cytoplasmic retention was driven by an Src inhibitor. Indeed, integrin α5β1-Src signalling plays a crucial role in different cancer types,^45–47^ and activated Src is capable of inducing YAP dephosphorylation and nuclear accumulation to regulate the downstream target gene expression.^48–50^ These results indicate that activated Src, but not FAK, may be partially responsible for TNC/integrin α5β1-mediated YAP activation in ES. Moreover, MYC was downregulated (Fig. 6a) in parallel with high levels of YAP phosphorylation (Fig. 5a) after TNC depletion in A673 cells. MYC, which was reported to be a transcriptional target of YAP in both gastric cancer and osteosarcoma,^51,52^ can transcriptionally activate MALAT1 by directly binding to the MALAT1 promoter region.^36^ These results indicate that MYC may be partly responsible for YAP-mediated MALAT1 activation.

In summary, our study demonstrates that MALAT1 upregulation induced by integrinα5β1-mediated YAP activation may be responsible for TNC-regulated tumour progression in ES and that TNC overexpression is associated with poor survival in ES patients. These findings may provide new insights into the role of TNC in the treatment and prognosis of ES.

## Supplementary information

Supplementary materials

## Data Availability

All the data analysed or generated in this study are included in this article and its supplementary files.

## References

[1] Pedersen EA, Menon R, Bailey KM, Thomas DG, Van Noord RA, Tran J (2016). Activation of Wnt/beta-Catenin in Ewing sarcoma cells antagonizes EWS/ETS function and promotes phenotypic transition to more metastatic cell states. Cancer Res..

[2] Lawlor ER, Sorensen PH (2015). Twenty years on: what do we really know about Ewing sarcoma and what is the path forward?. Crit. Rev. Oncog..

[3] Wan W, Lou Y, Hu Z, Wang T, Li J, Tang Y (2017). Factors affecting survival outcomes of patients with non-metastatic Ewing’s sarcoma family tumors in the spine: a retrospective analysis of 63 patients in a single center. J. Neurooncol.

[4] Gaspar N, Di Giannatale A, Geoerger B, Redini F, Corradini N, Enz-Werle N (2012). Bone sarcomas: from biology to targeted therapies. Sarcoma.

[5] Gaspar N, Hawkins DS, Dirksen U, Lewis IJ, Ferrari S, Le Deley MC (2015). Ewing Sarcoma: current management and future approaches through collaboration. J. Clin. Oncol..

[6] Erkizan HV, Kong Y, Merchant M, Schlottmann S, Barber-Rotenberg JS, Yuan L (2009). A small molecule blocking oncogenic protein EWS-FLI1 interaction with RNA helicase A inhibits growth of Ewing’s sarcoma. Nat. Med.

[7] Riggi N, Knoechel B, Gillespie SM, Rheinbay E, Boulay G, Suva ML (2014). EWS-FLI1 utilizes divergent chromatin remodeling mechanisms to directly activate or repress enhancer elements in Ewing sarcoma. Cancer Cell.

[8] Yoshida T, Akatsuka T, Imanaka-Yoshida K (2015). Tenascin-C and integrins in cancer. Cell Adh. Migr..

[9] Sun Z, Schwenzer A, Rupp T, Murdamoothoo D, Vegliante R, Lefebvre O (2018). Tenascin-C promotes tumor cell migration and metastasis through Integrin alpha9beta1-mediated YAP inhibition. Cancer Res.

[10] San Martin R, Pathak R, Jain A, Jung SY, Hilsenbeck SG, Pina-Barba MC (2017). Tenascin-C and Integrin alpha9 mediate interactions of prostate cancer with the bone microenvironment. Cancer Res..

[11] Zhang Z, Yu B, Gu Y, Zhou S, Qian T, Wang Y (2016). Fibroblast-derived tenascin-C promotes Schwann cell migration through beta1-integrin dependent pathway during peripheral nerve regeneration. Glia.

[12] Katoh D, Nagaharu K, Shimojo N, Hanamura N, Yamashita M, Kozuka Y (2013). Binding of alphavbeta1 and alphavbeta6 integrins to tenascin-C induces epithelial-mesenchymal transition-like change of breast cancer cells. Oncogenesis.

[13] Rupp T, Langlois B, Koczorowska MM, Radwanska A, Sun Z, Hussenet T (2016). Tenascin-C orchestrates glioblastoma angiogenesis by modulation of pro- and anti-angiogenic signaling. Cell Rep..

[14] Oskarsson T, Acharyya S, Zhang XH, Vanharanta S, Tavazoie SF, Morris PG (2011). Breast cancer cells produce tenascin C as a metastatic niche component to colonize the lungs. Nat. Med.

[15] Sarkar S, Mirzaei R, Zemp FJ, Wei W, Senger DL, Robbins SM (2017). Activation of NOTCH Signaling by tenascin-C promotes growth of human brain tumor-initiating cells. Cancer Res..

[16] Saupe F, Schwenzer A, Jia Y, Gasser I, Spenle C, Langlois B (2013). Tenascin-C downregulates wnt inhibitor dickkopf-1, promoting tumorigenesis in a neuroendocrine tumor model. Cell Rep..

[17] Lei L, Chen J, Huang J, Lu J, Pei S, Ding S (2018). Functions and regulatory mechanisms of metastasis-associated lung adenocarcinoma transcript 1. J. Cell Physiol..

[18] Fan Y, Shen B, Tan M, Mu X, Qin Y, Zhang F (2014). TGF-beta-induced upregulation of malat1 promotes bladder cancer metastasis by associating with suz12. Clin. Cancer Res.

[19] Leyh M, Seitz A, Durselen L, Schaumburger J, Ignatius A, Grifka J (2014). Subchondral bone influences chondrogenic differentiation and collagen production of human bone marrow-derived mesenchymal stem cells and articular chondrocytes. Arthritis Res Ther..

[20] Shalem O, Sanjana NE, Hartenian E, Shi X, Scott DA, Mikkelson T (2014). Genome-scale CRISPR-Cas9 knockout screening in human cells. Science.

[21] Sanjana NE, Shalem O, Zhang F (2014). Improved vectors and genome-wide libraries for CRISPR screening. Nat. Methods.

[22] Hollands C (1986). The Animals (scientific procedures) Act 1986. Lancet.

[23] Li Z, Xiao J, Wu X, Li W, Yang Z, Xie J (2012). Plumbagin inhibits breast tumor bone metastasis and osteolysis by modulating the tumor-bone microenvironment. Curr. Mol. Med.

[24] Ji Q, Zhang L, Liu X, Zhou L, Wang W, Han Z (2014). Long non-coding RNA MALAT1 promotes tumour growth and metastasis in colorectal cancer through binding to SFPQ and releasing oncogene PTBP2 from SFPQ/PTBP2 complex. Br. J. Cancer.

[25] Folpe AL, Goldblum JR, Rubin BP, Shehata BM, Liu W, Dei Tos AP (2005). Morphologic and immunophenotypic diversity in Ewing family tumors: a study of 66 genetically confirmed cases. Am. J. Surg. Pathol..

[26] Kim SK, Park YK (2016). Ewing sarcoma: a chronicle of molecular pathogenesis. Hum. Pathol..

[27] Smith R, Owen LA, Trem DJ, Wong JS, Whangbo JS, Golub TR (2006). Expression profiling of EWS/FLI identifies NKX2.2 as a critical target gene in Ewing’s sarcoma. Cancer Cell.

[28] Wang J, Wang H, Zhang Y, Zhen N, Zhang L, Qiao Y (2014). Mutual inhibition between YAP and SRSF1 maintains long non-coding RNA, Malat1-induced tumourigenesis in liver cancer. Cell Signal.

[29] Behrem S, Zarkovic K, Eskinja N, Jonjic N (2005). Distribution pattern of tenascin-C in glioblastoma: correlation with angiogenesis and tumor cell proliferation. Pathol. Oncol. Res..

[30] Gorthi A, Romero JC, Loranc E, Cao L, Lawrence LA, Goodale E (2018). EWS-FLI1 increases transcription to cause R-loops and block BRCA1 repair in Ewing sarcoma. Nature.

[31] Watanabe G, Nishimori H, Irifune H, Sasaki Y, Ishida S, Zembutsu H (2003). Induction of tenascin-C by tumor-specific EWS-ETS fusion genes. Genes Chromosomes Cancer.

[32] Xu Y, Zhang X, Hu X, Zhou W, Zhang P, Zhang J (2018). The effects of lncRNA MALAT1 on proliferation, invasion and migration in colorectal cancer through regulating SOX9. Mol. Med.

[33] Chen Y, Huang W, Sun W, Zheng B, Wang C, Luo Z (2018). LncRNA MALAT1 Promotes cancer metastasis in osteosarcoma via activation of the PI3K-Akt signaling pathway. Cell Physiol. Biochem.

[34] Sun Z., Ou C., Liu J., Chen C., Zhou Q., Yang S. et al. YAP1-induced MALAT1 promotes epithelial-mesenchymal transition and angiogenesis by sponging miR-126-5p in colorectal cancer. *Oncogene* **38**, 2627–2644 (2018) 10.1038/s41388-018-0628-y PMC6484768 30531836

[35] Halder G, Dupont S, Piccolo S (2012). Transduction of mechanical and cytoskeletal cues by YAP and TAZ. Nat. Rev. Mol. Cell Biol..

[36] Sun H, Lin DC, Cao Q, Pang B, Gae DD, Lee VKM (2017). Identification of a novel SYK/c-MYC/MALAT1 signaling pathway and its potential therapeutic value in Ewing sarcoma. Clin. Cancer Res..

[37] Roman J, Ritzenthaler JD, Roser-Page S, Sun X, Han S (2010). alpha5beta1-integrin expression is essential for tumor progression in experimental lung cancer. Am. J. Respir. Cell Mol. Biol..

[38] Caswell PT, Spence HJ, Parsons M, White DP, Clark K, Cheng KW (2007). Rab25 associates with alpha5beta1 integrin to promote invasive migration in 3D microenvironments. Dev. Cell.

[39] Kita D, Takino T, Nakada M, Takahashi T, Yamashita J, Sato H (2001). Expression of dominant-negative form of Ets-1 suppresses fibronectin-stimulated cell adhesion and migration through down-regulation of integrin alpha5 expression in U251 glioma cell line. Cancer Res..

[40] Huang W, Chiquet-Ehrismann R, Moyano JV, Garcia-Pardo A, Orend G (2001). Interference of tenascin-C with syndecan-4 binding to fibronectin blocks cell adhesion and stimulates tumor cell proliferation. Cancer Res..

[41] Loike JD, Cao L, Budhu S, Hoffman S, Silverstein SC (2001). Blockade of alpha 5 beta 1 integrins reverses the inhibitory effect of tenascin on chemotaxis of human monocytes and polymorphonuclear leukocytes through three-dimensional gels of extracellular matrix proteins. J. Immunol..

[42] Orend G, Huang W, Olayioye MA, Hynes NE, Chiquet-Ehrismann R (2003). Tenascin-C blocks cell-cycle progression of anchorage-dependent fibroblasts on fibronectin through inhibition of syndecan-4. Oncogene.

[43] Jachetti E, Caputo S, Mazzoleni S, Brambillasca CS, Parigi SM, Grioni M (2015). Tenascin-C protects cancer stem-like cells from immune surveillance by arresting T-cell activation. Cancer Res..

[44] Tanaka R, Seki Y, Saito Y, Kamiya S, Fujita M, Okutsu H (2014). Tenascin-C-derived peptide TNIIIA2 highly enhances cell survival and platelet-derived growth factor (PDGF)-dependent cell proliferation through potentiated and sustained activation of integrin alpha5beta1. J. Biol. Chem..

[45] Xiao Y, Li Y, Tao H, Humphries B, Li A, Jiang Y (2018). Integrin alpha5 down-regulation by miR-205 suppresses triple negative breast cancer stemness and metastasis by inhibiting the Src/Vav2/Rac1 pathway. Cancer Lett..

[46] Hamurcu Z, Delibasi N, Gecene S, Sener EF, Donmez-Altuntas H, Ozkul Y (2018). Targeting LC3 and Beclin-1 autophagy genes suppresses proliferation, survival, migration and invasion by inhibition of cyclin-D1 and uPAR/integrin beta1/ Src signaling in triple negative breast cancer cells. J. Cancer Res Clin. Oncol..

[47] Kim YJ, Jung K, Baek DS, Hong SS, Kim YS (2017). Co-targeting of EGF receptor and neuropilin-1 overcomes cetuximab resistance in pancreatic ductal adenocarcinoma with integrin beta1-driven Src-Akt bypass signaling. Oncogene.

[48] Si Y, Ji X, Cao X, Dai X, Xu L, Zhao H (2017). Src inhibits the hippo tumor suppressor pathway through tyrosine phosphorylation of Lats1. Cancer Res..

[49] Kakae K, Ikeuchi M, Kuga T, Saito Y, Yamaguchi N, Nakayama Y (2017). v-Src-induced nuclear localization of YAP is involved in multipolar spindle formation in tetraploid cells. Cell Signal.

[50] Elbediwy A, Vincent-Mistiaen ZI, Spencer-Dene B, Stone RK, Boeing S, Wculek SK (2016). Integrin signalling regulates YAP and TAZ to control skin homeostasis. Development.

[51] Choi W, Kim J, Park J, Lee DH, Hwang D, Kim JH (2018). YAP/TAZ Initiates gastric tumorigenesis via upregulation of MYC. Cancer Res.

[52] Shen Y, Zhao S, Wang S, Pan X, Zhang Y, Xu J (2019). S1P/S1PR3 axis promotes aerobic glycolysis by YAP/c-MYC/PGAM1 axis in osteosarcoma. EBioMedicine.

