# Supplementary material for: EWS-FLI1-mediated tenascin-C expression promotes tumour progression by targeting MALAT1 through integrin α5β1-mediated YAP activation in Ewing sarcoma
Source: Br J Cancer. 2019 Oct 25;121(11):922–33. doi: 10.1038/s41416-019-0608-1 (PMC6889507; doi:10.1038/s41416-019-0608-1)
Supplement: Supplementary file 1 — Supplementary materials [file 41416_2019_608_MOESM1_ESM.docx]

**Supplementary materials**


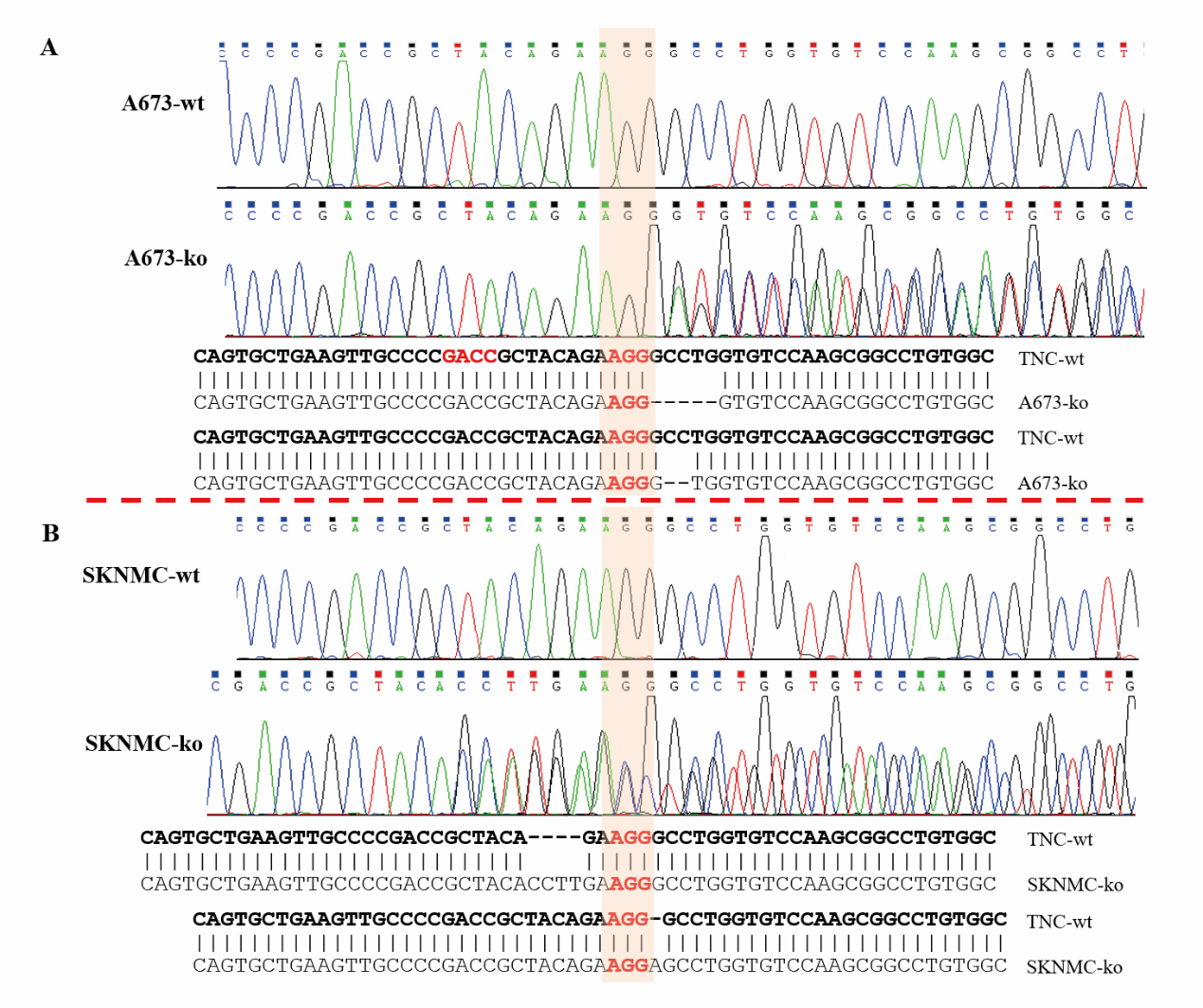


**Supplementary Fig. 1 DNA sequencing of the TNC gene in TNC-ko A673 and SKNMC cell lines.**

**Supplementary Table 1 The list of primary and secondary antibodies used in this study**

| **Antibody** | **Product code** | **Company** | **Application** | **Species** |
| --- | --- | --- | --- | --- |
| Anti-Tenascin-C | ab108930 | Abcam | IHC, WB | Rabbit |
| Anti-CD99  Anti-YAP  Anti-pYAP | ab75858  ab52771  ab76252 | Abcam  Abcam  Abcam | IHC  ICC, WB  WB | Rabbit  Rabbit  Rabbit |
| Anti-Cyr61 | ab24448 | Abcam | WB | Rabbit |
| Anti-CTGF | ab231824 | Abcam | WB | Rabbit |
| Anti-SRSF1 | ab38017 | Abcam | WB | Rabbit |
| Anti-Flag | ab205606 | Abcam | WB | Rabbit |
| Anti-Integrin α2 | ab133557 | Abcam | WB | Rabbit |
| Anti-Integrin α5 | ab150361 | Abcam | WB | Rabbit |
| Anti-Integrin α7 | ab182941 | Abcam | WB | Rabbit |
| Anti-Integrin α8 | ab106322 | Abcam | WB | Rabbit |
| Anti-Integrin α9 | ab140599 | Abcam | WB | Rabbit |
| Anti-Integrin αV | ab179475 | Abcam | WB | Rabbit |
| Anti-Integrin β1 | ab179471 | Abcam | WB | Rabbit |
| Anti-Integrin β3 | ab197662 | Abcam | WB | Rabbit |
| Anti-Integrin β6 | ab187155 | Abcam | WB | Rabbit |
| Anti-Notch1 | ab52627 | Abcam | WB | Rabbit |
| Anti-Notch2 | ab8926 | Abcam | WB | Rabbit |
| Anti-Notch3 | ab23426 | Abcam | WB | Rabbit |
| Anti-Notch4 | sc-393893 | SantaCruz | WB | Mouse |
| Anti-EGFR  Anti-Src  Anti-pSrc  Anti-FAK  Anti-pFAK  Anti-Actin  Normal Rabbit IgG  HRP-antiRabbit IgG  HRP-antiMouse IgG  FITC-antiRabbit IgG | ab52894  ab109381  #6943  ab40794  ab81298  ab8227  ab172730  GB23303  GB23301  GB22303 | Abcam  Abcam  CST  Abcam  Abcam  Abcam  Abcam  Servicebio  Servicebio  Servicebio | WB  WB  WB  WB  WB  WB  WB, ICC  IHC, WB  WB  ICC | Rabbit  Rabbit  Rabbit  Rabbit  Rabbit  Rabbit  Rabbit  Goat  Goat  Goat |
| Anti-EWSR1/EWS | ab133288 | Abcam | WB | Rabbit |
| Anti-FLI1 | ab15289 | Abcam | WB, ChIP | Rabbit |
| Anti-Syk | #2712 | CST | WB | Rabbit |
| Anti-pSyk | #2710 | CST | WB | Rabbit |
| Anti-MYC | Ab32152 | Abcam | WB | Rabbit |

**Supplementary Table 2 The qRT-PCR primers used in the study**

| **Gene** | **Forward** | **Reverse** |
| --- | --- | --- |
| Integrin α2 | GGACTTTCGCATCATCAACG | CTTCGGCTTTCTCATCAGGT |
| Integrin α5 | CAGCCCTACATTATCAGAGCAA | GTTCACGGCAAAGTAGTCACAG |
| Integrin α7 | CGAGATTTCCCTTGCATTCG | AGTCAGTCTCCTCCAGGCTCAA |
| Integrin α8 | TTTGGGAGATTCGGTAGTGC | AACTTGGGAAGGCTTGGTGT |
| Integrin α9 | GAACCCAGAAGAGGTGACGG | CAGCAGCAGGAAGATGAGGA |
| Integrin αV | GTGGACAGTCCTGCCGAGTA | TGTTTCGACCTCACAGATGC |
| Integrin β1 | GAGATGGGAAACTTGGTGGC | GACTTCTGAGGAAAGGGAAT |
| Integrin β3 | GCCACGTCTACCTTCACCAA | GACATTCTCCCAACCTACCC |
| Integrin β6 | ATTGATCTTCGCTGTAACCC | TCAGACCGCAGTTCTTCATA |
| Notch1 | GCCGTCATCTCCGACTTCATCT | GGGTGTCTCCTCCCTGTTGTTCT |
| Notch2 | GACCCTGTCATACCCTCTTGT | CATGCTTACGCTTTCGTTTT |
| Notch3 | ATTCTCATCCGAAACCGCTCTAC | GGGTCTCCTCCTTGCTATCCTG |
| Notch4 | ATAATGCGAGGAAGATACGGAGTG | AGGCGGGATCGGAATGTTGG |
| EGFR | GCCAAGGCACGAGTAACAAG | AGGGCAATGAGGACATAACCAG |
| MALAT1 | ACAGTGCAGCTTTGGTTCAT | GGTACACCCAGTGGCTCATA |
| TNC | CCTTGCTGTAGAGGTCGTCA | CCAACCTCAGACACGGCTA |

**Supplementary Table 3 The primers used in the ChIP assay**

| **Site** | **Forward** | **Reverse** |
| --- | --- | --- |
| 1: -1442~-1447 | ATGGGCTTTCTTGCTGTCTG | ATGGAGGTCTCAATTATCCACTTA |
| 2: -748~-753 | TGCTACTTCTCCACTCCCTT | AACCTAATCCTTGAGCCATAA |
| 3: -536~-541 | CAGGCACAGGGCATGAACAA | CGTGCGGGACGCTCACATCA |
| 4: -360~-368 | CCCCACCTGCTGAGACCTAA | TCTAATTGTGAGACGGATGCC |
| 5: -121~-129 | CCAGGCATCCGTCTCACAAT | CCAGACATCCTTTCCCACTTTT |
